# Loop and Bridge Conformations of ABA Triblock Comb Copolymers: A Conformational Assessment for Molecular Composites

**DOI:** 10.3390/polym14112301

**Published:** 2022-06-06

**Authors:** Jihoon Park, Je-Yeon Jung, Hyun-Woo Shin, Jong-Wan Park, Joona Bang, June Huh

**Affiliations:** 1 Department of Chemical and Biological Engineering, Korea University, Seoul 02841, Korea; ayanami9306@korea.ac.kr (J.P.); jyjung17@korea.ac.kr (J.-Y.J.); 2 College of Medicine, Seoul National University, Seoul 03080, Korea; charlie@snu.ac.kr (H.-W.S.); parkjw@snu.ac.kr (J.-W.P.); 3 Department of Life Sciences, Korea University, Seoul 02841, Korea

**Keywords:** triblock copolymer, comb polymer, bridge conformation, loop conformation

## Abstract

We computationally investigate the conformational behavior, “bridging” chain, between different the phase-separated domains vs “looping” chain on the same domain, for two chain architectures of ABA triblock copolymers, one with a linear architecture (L-TBC) and the other with comb architecture (C-TBC) at various segregation regimes using dissipative particle dynamics (DPD) simulations. The power-law relation between the bridge fraction (Φ) and the interaction parameter (χ) for C-TBC is found to be $\Phi ∼\chi^{-1.6}$ in the vicinity of the order-disorder transition ($\chi_{ODT}$), indicating a drastic conversion from the bridge to the loop conformation. When χ further increases, the bridge-loop conversions slow down to have the power law, $\Phi ∼\chi^{-0.18}$, approaching the theoretical power law $\Phi ∼\chi^{-1/9}$ predicted in the strong segregation limit. The conformational assessment conducted in the present study can provide a strategy of designing optimal material and processing conditions for triblock copolymer either with linear or comb architecture to be used for thermoplastic elastomer or molecular nanocomposites.

## 1. Introduction

**Figure 1:**
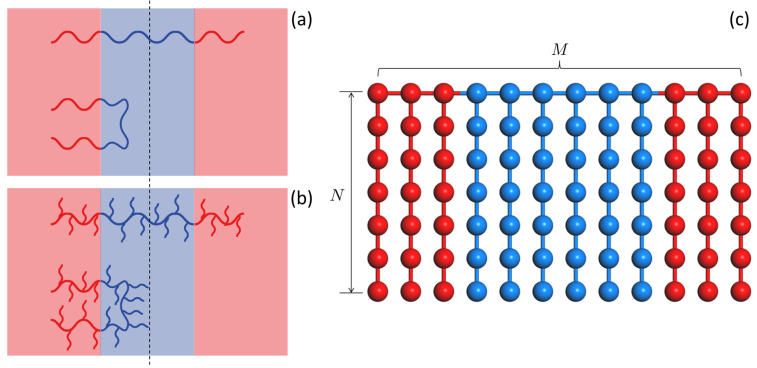
Illustration of (**a**) bridge and loop conformation for ABA linear triblock copolymer (L-TBC), (**b**) those of ABA comb triblock copolymer (C-TBC) in the phase-separated domains, and (**c**) the chain architecture of C-TBC investigated in this study. In (**a,b**), the red-colored regions and the blue-colored region are the A- and the B-domain, respectively, and the dashed line in the B-domain represents the midplane.

Triblock copolymer (TBC), comprised of three polymer blocks linearly linked together in either ABA or ABC form, is an industrially important polymer used in a wide range of fields such as thermoplastic elastomer [1,2,3,4,5,6] and molecular composites, refs. [7,8,9] where the conformational behavior of TBC is often a key factor for determining the resultant performance of such applications. For instance, well-designed TBC consisting of two terminal blocks with hard segments and a middle block with rubbery segments can form hard nanodomains embedded in rubbery matrix, where chains bridging between two different hard domains, which can efficiently persist the mechanical deformation, play a critical role for the overall mechanical properties of thermoplastic elastomer or molecular composite systems [10,11,12]. For this reason, there have been steady interest in the chain conformation of TBC with respect to the preferred conformation, “bridging” between different domains vs “looping” on the same domain (Figure 1). Previous theoretical approach based on the self-consistent theory predicted the fraction of bridge conformation (Φ) scale as $\Phi ∼{\left(\chi N\right)}^{-1/9}$ [13] in the limit of strong segregation where χ is the Flory–Huggins interaction parameter between unlike segments and *N* is the degree of polymerization of TBC. The assessment of bridging fraction have been reported by several groups. Earlier experimental works reported the loop/bridge conformation ratios of TBC determined from viscoelastic, dielectric or mechanical behavior [12,14,15,16]. In the theoretical side, some computational works based on self-consistent field theory or coarse-grained simulations were also carried out to investigate the bridging fraction of the ordered TBC [13,15,17,18,19,20,21,22,23].

Comb copolymers, where two or more dissimilar types of side chains as macromers are grafted to a linear polymer backbone with a certain macromer sequence, have attracted much interest recently owing to their intriguing self-assembly behaviors [24,25,26,27,28,29]. Analogous to TBC having a triblock sequence based on monomeric units, comb copolymers can also have triblock sequence based on macromer units (Figure 1), which envisions its use for a novel type of molecular composites or thermoplastic elastomer [30,31,32] with a number of additional advantages such as more variety of options for functional design of macromers as a reinforcing component. However, despite growing interest in comb copolymers as a novel type of molecular composite materials, their conformational behavior, in particular, bridge vs loop conformations have not yet been investigated. In this brief report, as the first investigation of bridge conformation of comb copolymer, we report a simulation assessment of a bridge fraction of comb copolymer consisting of A and B macromers with an ABA triblock sequence (hereafter referred to as C-TBC) in a molten state focusing on its dependence on χ in the various segregation regime.

## 2. Simulation Methods

All simulations were carried out by a dissipative particle dynamics (DPD) [33,34] using the HOOMD package [35]. In the DPD model, polymers are represented by bead-spring chains, [36,37] where each bead representing a statistical monomer interacts with each other via a pairwise additive force. The force $f_{i}$ acting on bead *i* of mass $m_{i}$ at a position vector $r_{i}$ is given as:

(1) $$\begin{array}{c} f_{i}=m_{i}{\ddot{r}}_{i}=\sum_{j\neq i}\left(F_{ij}^{\left(C\right)}+F_{ij}^{\left(D\right)}+F_{ij}^{\left(R\right)}+F_{ij}^{\left(S\right)}\right), \end{array}$$

where $F_{ij}^{\left(C\right)}$, $F_{ij}^{\left(D\right)}$, $F_{ij}^{\left(R\right)}$, and $F_{ij}^{\left(S\right)}$ are a conservative force, a drag force, a random force, and spring force between bead *i* and *j*, respectively. The conservative force $F_{ij}^{\left(C\right)}$ is modeled as a soft core repulsion,

(2) $$\begin{array}{c} F_{ij}^{\left(C\right)}=\left\{\begin{array}{cc} a_{ij}\left(1-\frac{r_{ij}}{R_{c}}\right){\hat{r}}_{ij} & \mathrm{for}\;r_{ij}<R_{c}\\ 0 & \mathrm{otherwise}, \end{array}\right. \end{array}$$

where $a_{ij}$ is a maximum repulsion ($a_{ij}>0$) between beads *i* and *j*, $r_{ij}$ is the distance between bead *i* and *j*, ${\hat{r}}_{ij}$ is a unit vector along the direction from bead *i* to bead *j*, and $R_{c}$ is the cutoff distance. The drag force $F_{ij}^{\left(D\right)}$ and the random force $F_{ij}^{\left(R\right)}$ are given as:

(3) $$\begin{array}{cc} \; & F_{ij}^{\left(D\right)}=-\gamma {\left[w(r_{ij})\right]}^{2}\left({\hat{r}}_{ij}\cdot {\dot{r}}_{ij}\right){\hat{r}}_{ij} \end{array}$$

(4) $$\begin{array}{cc} \; & F_{ij}^{\left(R\right)}=\zeta_{ij}\left(t\right)w\left(r_{ij}\right)\sqrt{\frac{6k_{B}T\gamma }{\delta t}}{\hat{r}}_{ij}, \end{array}$$

where γ is the friction coefficient, $w(r_{ij})$ is a weight function related to $r_{ij}$, $\zeta_{ij}$ is a random number uniformly distributed in the range of [−1,1] generated independently for each pair of bead *i* and *j* at each time step, $k_{B}T$ is thermal energy, and δt is the time step size. Equations (3) and (4) ensure the consistency between kinetic energy and thermal energy via the amplitude of random noise ($\sqrt{6k_{B}T\gamma /\delta t}$), refs. [33,38] with the weight function *w* chosen to have the following form:

(5) $$\begin{array}{c} w\left(r\right)=\left\{\begin{array}{cc} 1-\frac{r}{R_{c}} & \mathrm{for}\;r<R_{c}\\ 0 & \mathrm{otherwise}. \end{array}\right. \end{array}$$

The bonding between bead *i* and *j*, responsible for chain connectivity, is taken into account by a spring force, $F_{ij}^{\left(S\right)}$:

(6) $$\begin{array}{c} F_{ij}^{\left(S\right)}=-K\left(r_{ij}-r_{o}\right){\hat{r}}_{ij}, \end{array}$$

where *K* is the spring constant and $r_{o}$ is the equilibrium bond length. The equation of motions (Equation (1)) for beads in the system were time-integrated using velocity-Verlet algorithm [39]:

(7) $$\begin{array}{cc} \; & r_{i}\left(t+\delta t\right)=r_{i}\left(t\right)+{\dot{r}}_{i}\left(t\right)\delta t+\frac{1}{2}{\ddot{r}}_{i}\left(t\right)\delta t^{2} \end{array}$$

(8) $$\begin{array}{cc} \; & {\dot{r}}_{i}\left(t+\delta t\right)={\dot{r}}_{i}\left(t\right)+\frac{1}{2}{\ddot{r}}_{i}\left(t\right)\delta t+\frac{1}{2}{\ddot{r}}_{i}\left(t+\delta t\right)\delta t. \end{array}$$

The basic units for length, mass, energy, and time in the simulation are set to be $R_{c}=1$, m=1, $k_{B}T=1$, and $t=R_{c}\sqrt{m/k_{B}T}=1$, respectively, and the time step δt is set to be δt=0.01 which is specified from the unit thermal energy, $k_{B}T=1$, for the consistency between thermal and kinetic energy. All DPD parameters introduced Equations (11)–(15) are rescaled according to these basic units, which are listed in Table 1.

Using the bead-spring chain model by DPD, architecturally monodisperse C-TBCs were generated in a $40R_{c}\times 40R_{c}\times 40R_{c}$ simulation box with a number density of beads $\rho =3R_{c}^{-3}$ chosen for the molten state. An ABA-type C-TBC chain consists of a backbone of *M* beads where A-macromers each with N−1 beads are grafted to the backbone in the two terminal backbone region each with M/4 beads, respectively, and B macromers each with N−1 beads are grafted to the backbone in the middle backbone region with M/2 beads. The periodic boundary conditions were applied in all axes of the simulation box. The unfavorable interaction between the beads of type A and type B was modeled using the maximum repulsion $a_{ij}$ introduced in Equation (2), where its value is given from the Flory interaction parameter between A- and B-bead, χ, using the relation, $c\chi =\Delta a/k_{B}T$, where $\Delta a=a_{AB}-\left(a_{AA}+a_{BB}\right)/2$ and the density-dependent parameter *c* is given as c=3.27 for the present choice of bead density [34]. The maximum repulsion between the same kind of beads is set to be $a_{AA}=a_{BB}=25k_{B}T/R_{c}$ and that between A and B beads, $a_{AB}$, is given according to a desired χ. The ordered state of each system was obtained by stepwise-increasing $\Delta a/k_{B}T$ from an athermal state ($\Delta a/k_{B}T=0.0$) to a desired $\Delta a/k_{B}T$ with the simulated annealing scheme [40] where the thermal profile ($\tau \equiv k_{B}T/\Delta a$) with time *t* is given by:

(9) $$\begin{array}{c} \tau (t+\Delta t)=\tau (t)-k(\tau (t)). \end{array}$$

Here, k(τ(t)) is the cooling schedule, i.e., the change of τ in the time interval $\Delta t=4\times 10^{4}\delta t$, which has the form of:

(10) $$\begin{array}{c} k\left(\tau \left(t\right)\right)=\left\{\begin{array}{cc} k_{o} & \mathrm{for}\;0.8\tau_{{\;}_{ODT}}<\tau \left(t\right)<2.5\tau_{{\;}_{ODT}}\\ 10k_{o} & \mathrm{otherwise}, \end{array}\right. \end{array}$$

where $\tau_{{\;}_{ODT}}$ is the τ at the ODT and $k_{o}$ is the cooling rate in the region nearby ODT. The value of $k_{o}$ in this study is set to be in the range of 0.003–0.006 depending on the statistical inefficiency analyzed for the given system [41].

Having obtained the ordered structures at desired $\Delta a/k_{B}T$, the systems were further equilibrated at each of $\Delta a/k_{B}T$ for $2\times 10^{6}\delta t$ followed by the production step for $2\times 10^{6}\delta t$ to produce configuration samples for thermodynamic average.

## 3. Results

We consider a symmetric ABA-type C-TBC chain architecture comprised of two terminal blocks each with M/4 A-macromers and a middle block with M/2 B-macromers where each macromer consists of N−1 beads (Figure 1c).

**Figure 2:**
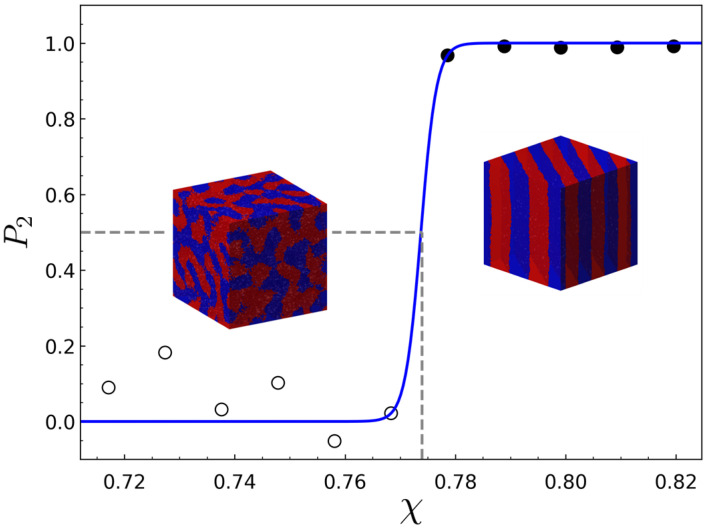
The order parameter versus the interaction parameter for the C-TBC with {M=24,N=4}. The open circles and filled circles represent the points where the disordered and the ordered phases are stable, respectively, and the blue solid line is fit to a three-parameter sigmoidal function. The inset images show the two example structures simulated at the disordered and ordered region.

It is noted that the the C-TBC chain with N=1 reduces to the ABA triblock chain with linear architecture (hereafter referred to as L-TBC) whose conformational behavior is to be compared to that of comb architecture, i.e., C-TBC with N≠1. To investigate the conformational behavior of C-TBC chain in the disordered and ordered states, we first located the order–disorder transition (ODT) of molten C-TBC from the DPD simulations. To do this, the density fluctuation of A-bead and B-bead in the systems simulated at a desired χ was analyzed using scattering function, computed by:

(11) $$\begin{array}{c} S\left(q\right)=\frac{1}{V}\langle \sum_{i<j}e^{iq\cdot (r_{i}-r_{j})}\Psi_{i}\Psi_{j}\rangle , \end{array}$$

where q is the wave vector, *V* is the volume of the system, $r_{i}$ is the coordinates of the bead *i*, $\Psi_{i}$ is the occupation variable having values of −1 or 1 if the bead *i* is an A bead or a B bead, respectively, and the bracket   indicates a thermodynamic average. The order parameter was then used for the determination of ODT, which can be computed by the second order Legendre polynomial using the scattering function, $P_{2}=\frac{3}{2}\sum_{q}{\left(\hat{q}\cdot {\hat{q}}_{1}\right)}^{2}S\left(q\right)/\sum_{q}S\left(q\right)-1$ where $\hat{q}$ and ${\hat{q}}_{1}$ are the unit vectors in the direction of a wave vector q and in the direction of the dominant wave vector $q_{1}$, respectively. In the disordered state where $\chi <\chi_{ODT}$, the density fluctuations of A-beads, described by wave vector q, are broadly distributed and therefore the order parameter $P_{2}$ fluctuates around zero. When the ordered phase is formed such that $\chi >\chi_{ODT}$, a certain wave vector becomes dominant (i.e., S(q) is sharply peaked at the dominant wave vector $q_{1}$), which leads to the increase in $P_{2}$. Figure 2 shows an example of the determination of ODT by the order parameter, plotted against Δa for molten C-TBC with {M=24,N=4}. Table 2 summarizes the value of χ at ODT, $\chi_{{\;}_{ODT}}$ and the domain spacing, *L*, for the L-TBC and C-TBC systems simulated in this study. It should be noted that the values of *N* and *M* for L-TBC (N=1) and C-TBC (N≠1) samples were chosen such that the domain spacing in all copolymer samples are nearly the same to exclude the effect of domain size on the conformational behavior about the bridge and loop.

For measuring the fraction of bridge and loop conformation, we followed the method used in our previous work for L-TBC [20] where the bridge and loop fractions were estimated using the angle θ between two vectors,

(12) $$\begin{array}{c} \cos\theta =\frac{r_{BA}\cdot r_{BA^{\prime }}}{|r_{BA}\left|\right|r_{BA^{\prime }}|}, \end{array}$$

where $r_{BA}$ and $r_{BA^{\prime }}$ are the two vectors from the middle position B pointing to two terminal positions, A and A′, in the backbone, respectively. The fraction of bridge conformation, Φ, was then measured by the following formula,

(13) $$\begin{array}{c} \Phi =\int_{-1}^{0}P\left(u\right)du, \end{array}$$

where u≡cosθ and P(u) is the probability density function of *u*. The fraction of loop conformation is then given as 1−Φ.

**Figure 3:**
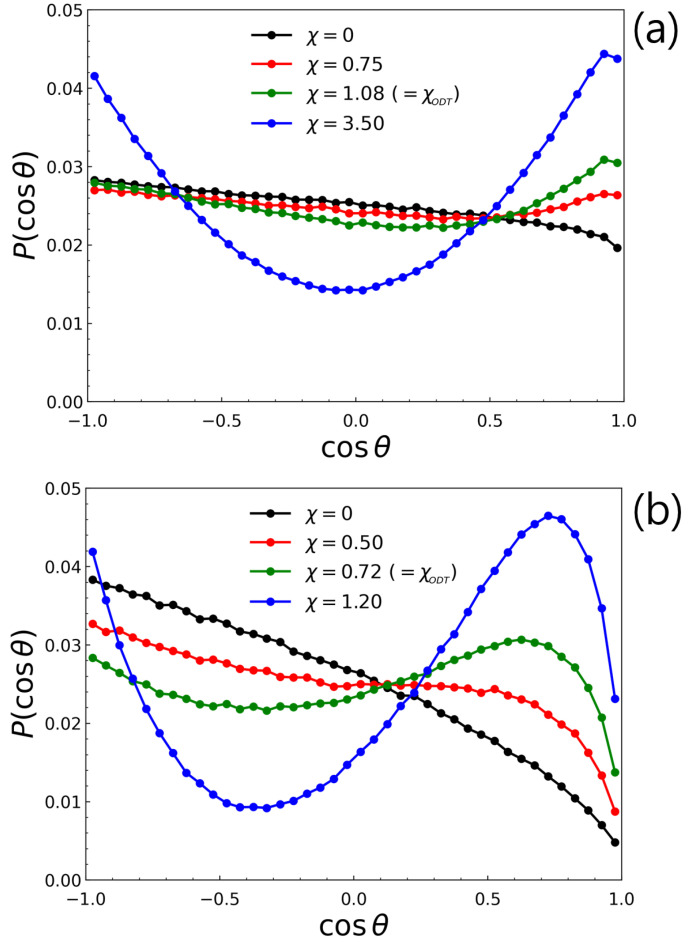
P(cosθ) for (**a**) L-TBC (i.e., N=1) with M=48 and for (**b**) C-TBC with M=16 and M=8 at various χ values. The $\chi_{ODT}$ for (**a**,**b**) were χ=1.08 and χ=0.72, respectively.

Figure 3a shows the behavior of P(cosθ) for L-TBC (i.e., N=1) with M=48 at athermal state (χ=0), $\chi <\chi_{ODT}$, $\chi =\chi_{ODT}$, and $\chi \gg \chi_{ODT}$. For chains satisfying random walk so that the two vectors pointing termini $r_{BA}$ and $r_{BA^{\prime }}$ are randomly oriented to each other, P(cosϕ) must follow a uniform distribution. However, because of excluded volume interactions, the distribution shows a slight slope even at athermal condition (χ=0) and rapidly decays at cosϕ≃1 where the two terminal A-blocks overlap with their radii of gyrations. The higher value of P(cosθ) at cosθ=0.5−1.0 at χ=0.75 when compared to that at χ=0 is due to the formation of longer-lived transient domain at χ=0.75. Thus, the system at χ=0.75 creates more bridge conformations, which is reflected by the higher value of P(cosθ) at cosθ=0.5−1.0. As the system becomes ordered ($\chi >\chi_{ODT}=0.83$), the shape of the distribution becomes parabolic, which indicates that the L-TBC chains adopt either bridge or loop conformation as the phase-separated domains are formed. Similar χ-evolution of the distribution but more pronounced shape (i.e., steeper slopes at χ=0 and more convex curves in the region of cosθ=0.5−1.0 at higher χ) were observed for C-TBC (Figure 3b), which reflect the more significant effect of excluded volume interactions owing to bulky macromers.

**Figure 4:**
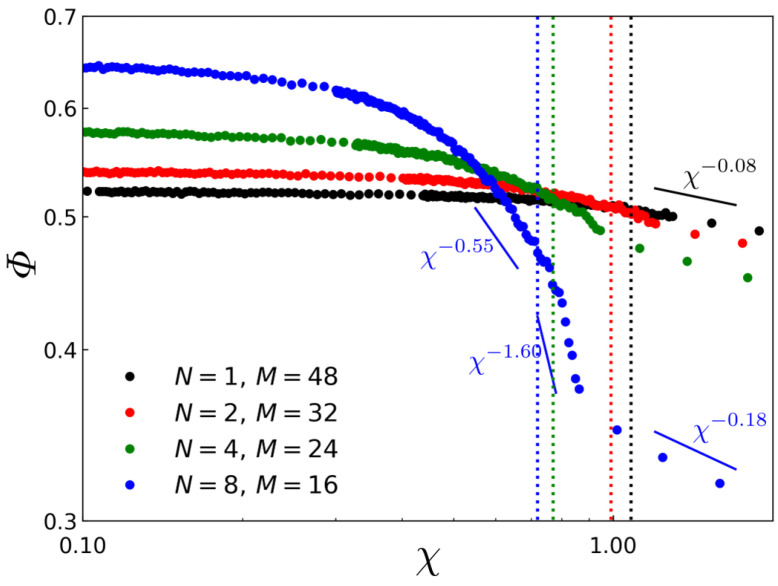
The fractions of bridge conformation, Φ, versus χ for the simulated L-TBC and C-TBC samples. The vertical dashed lines represent the $\chi_{ODT}$ for the corresponding copolymer samples.

In Figure 4, the fractions of bridge conformation, Φ, measured by Equations (12) and (13), are plotted as a function of χ for various L- and C-TBC systems. It is noted from Figure 4 that, as the system enters the ordered regime, the bridge fraction for L-TBC ({N=1,M=48}) and for C-TBC with relatively shorter side chains ({N=2,M=32}, {N=4,M=24}) scales similarly as $\chi^{-0.08}$, showing slightly smaller exponent than the strong segregation theory result, ν=1/9≃0.11 for $\chi^{-\nu }$, predicted for L-TBC [13]. An interesting result is found for C-TBC with relatively longer side chains ({N=8,M=16}) where the bridge fraction decreases rapidly as χ increases in the vicinity to $\chi_{ODT}$ but still in the disordered region, following the $\chi^{-0.55}$ power law. In this disordered region near ODT, long-lived but still transient A-domains are responsible for such uneven conformational behavior. The exponent becomes even larger when this C-TBC system enters the ordered regime showing a very rapid decay of $\Phi ∼\chi^{-1.6}$ followed by an another regime of slow conversion from loop to bridge, $\Phi ∼\chi^{-0.18}$. It is also of interest that the bridge conformation for C-TBC with a longer side chain is preferred over the loop conformation in the very low-χ regime (Φ≃0.65 at $\chi \ll \chi_{ODT}$) and becomes rapidly unfavored as χ passes $\chi_{ODT}$ (Φ≲0.35 at $\chi \gg \chi_{ODT}$), which is in contrast with the behavior of L-TBC showing Φ≃0.52 at $\chi \ll \chi_{ODT}$ and Φ≲0.5 at $\chi \gg \chi_{ODT}$. We interpret this conformational behavior of C-TBC as follows. In the very low χ regime, the loop conformation of C-TBC is less favored due to the steric repulsion between two terminal blocks consisting of A-macromers, resulting in a larger fraction of bridge conformation. As χ increases so that the transient domains at $\chi <\chi_{ODT}$ or the phase-separated domains at $\chi >\chi_{ODT}$ are formed, the bridge conformation starts to be less preferred, because the number of possible ways for backbone paths, which is obliged to be across the midplane of B-domain, is smaller than that of the loop conformation which does not have to pass through the midplane (See schematic in Figure 1a,b) [31,42]. This different restriction on bridge and loop conformation imposed by the phase-separated domains gives rise to the preference of the loop conformation over the bridge conformation as χ increases and this propensity becomes even more pronounced for comb architecture by its side chain which can save the stretching energy of looping C-TBC. In general, the domain spacing of the block copolymer system is a result of the balance between the minimal interfacial energy and the minimal stretching energy: the former wants to expand the domain spacing while the latter wants to compress the spacing. It can be noticed that the difference in backbone stretching energy between the loop conformation and the bridge conformation for C-TBC is larger than that for L-TBC because the stretching energy of backbone of a looping C-TBC can be saved more than that of a looping L-TBC due to the presence of side chains filling in the region nearby the midplane.

## 4. Conclusions

In conclusion, the computational assessments of bridge and loop conformation for two chain architectures of ABA triblock copolymers, one with a linear architecture (L-TBC) and the other with comb architecture (C-TBC), were performed at various segregation regimes using DPD simulations. It is found from DPD simulation that the bridge conformation of C-TBC favored in the very low χ regime is converted to the loop conformation much more rapidly than the bridge-loop conversion in L-TBC. The power law relation between the bridge fraction (Φ) and the interaction parameter (χ) for C-TBC is found to be $\Phi ∼\chi^{-1.6}$ in the ordered state but in the vicinity of $\chi_{ODT}$ indicating a drastic conversion from the bridge to the loop as the system enters the regime of the ordered state. When the segregation power becomes stronger such that $\chi \gg \chi_{ODT}$, the bridge–loop conversions slow down to the power law, $\Phi ∼\chi^{-0.18}$, presumably approaching the theoretical power law $\Phi ∼\chi^{-1/9}$ in the strong segregation limit. Assuming that a large amount of bridge conformation in the TBC system is a key requirement for the reliable mechanical performance of TBC materials to be used for thermoplastic elastomers or molecular composites, the finding for C-TBC in the present study is somewhat discouraging, suggesting that the prolonged equilibration of the C-TBC system could cause an undesirable TBC system containing the loop conformation dominant over bridge conformation. However, noting in C-TBC that the bridge conformation is dominant in the low χ regime, finding an efficient thermal profile for manipulating the C-TBC system can be a solution to detour the rapid bridge–loop conversion, which will be investigated in our future work.

## Figures and Tables

**Table 1:** The list of the DPD parameters used in the present study.

Parameter	Value	Unit ^1^	Equations
$a_{ii}\;\left(a_{AA},a_{BB}\right)$	25.0	$k_{B}T/R_{c}$	(2)
γ	4.5	$\sqrt{mk_{B}T}/R_{c}$	(3) and (4)
*K*	100.0	$k_{B}T/R_{c}^{2}$	(6)
$r_{o}$	0.7	$R_{c}$	(6)

^1^ The basic units for length, mass, and energy are set to be *R_c_* = 1, *m* = 1, and *k_B_T* = 1, respectively.

**Table 2:** The chain architectures of triblock copolymers simulated in this study and the list of the χ values at ODT, $\chi_{{\;}_{ODT}}$ and the domain spacing, *L*, for these triblock copolymers in molten state.

Architecure	*M*	*N*	$\chi_{{\;}_{\mathrm{ODT}}}$	*L* ^1^
L-TBC	48	1	1.08	8.26 ± 0.19
C-TBC	32	2	0.99	8.18 ± 0.26
C-TBC	24	4	0.77	8.17 ± 0.16
C-TBC	16	8	0.72	8.26 ± 0.18

^1^ measured at χ*MN* = 150.

## Data Availability

The data presented in this study are available on request from the corresponding author.
